# Supplementary material for: Challenging the Existing Model of the Hexameric HIV-1 Gag Lattice and MA Shell Superstructure: Implications for Viral Entry
Source: Viruses. 2021 Jul 31;13(8):1515. doi: 10.3390/v13081515 (PMC8402665; doi:10.3390/v13081515)
Supplement: Supplementary file 1 [file viruses-13-01515-s001.zip › viruses-1267917-supplementary.pdf]

Supplemental figures:

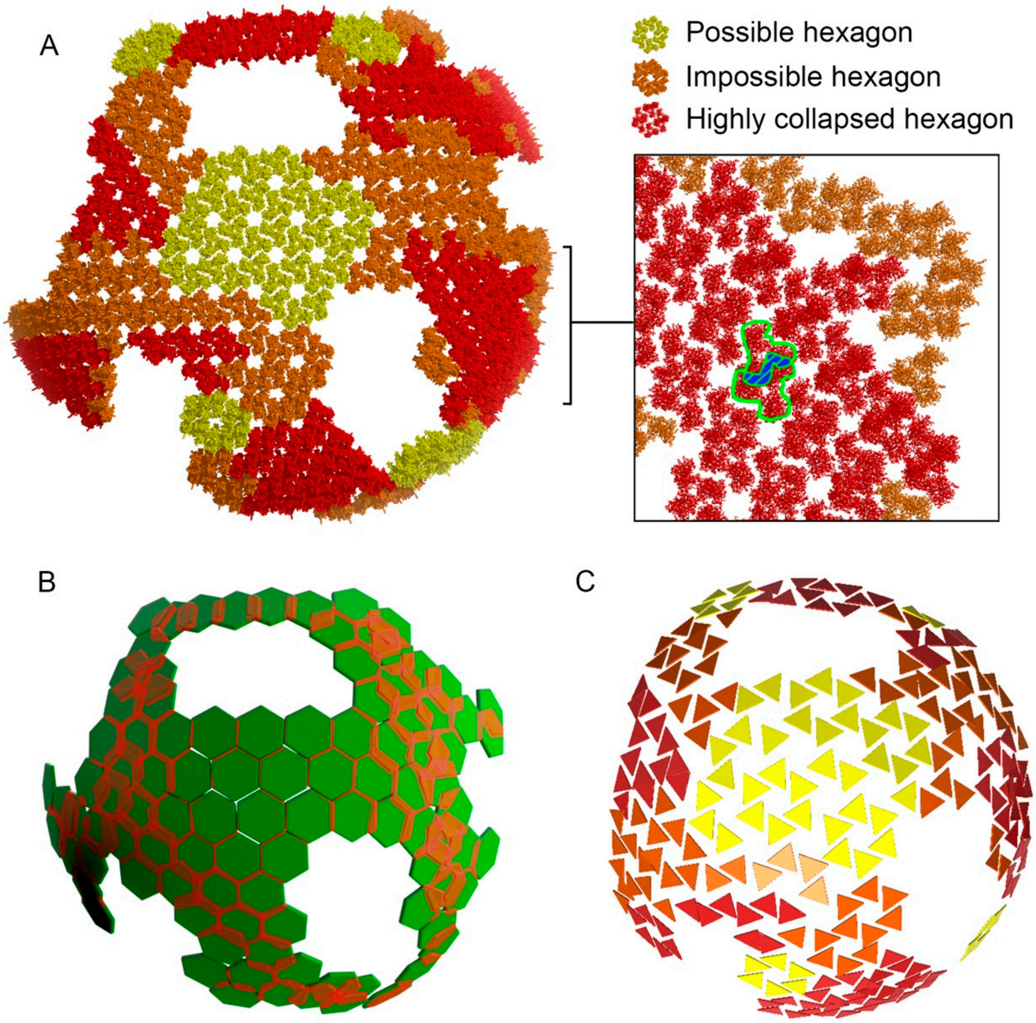

Supplemental Figure S1. Reconstruction and analysis of a typical quasi-equivalent MA lattice model. Model was obtained from a preprint by Qu et al. to illustrate the impossible existence of hexagonal MA lattices containing breaks (https://doi.org/10.1101/2020.09.23.309542). (A) A best-fit model was calculated to maximize the number of possible MA hexagons (yellow). Inevitably, adjacent MA trimers start to overlap with each other, creating impossible hexagonal lattices (orange). If further propagation of these patterns is continued, highly collapsed hexagons emerge as the best possible solution (red). Inset shows a zoomed side view and highlights two overlapping red MA trimers. (B) Regular green hexagon tiles highlight in red the overlapping needed to allow hexagons to fit the proposed model. (C) The use of triangles rather than hexagons makes it particularly difficult for readers to assess the magnitude of the geometrical incompatibility. Triangles are colour-coded as in panel A.

A

B

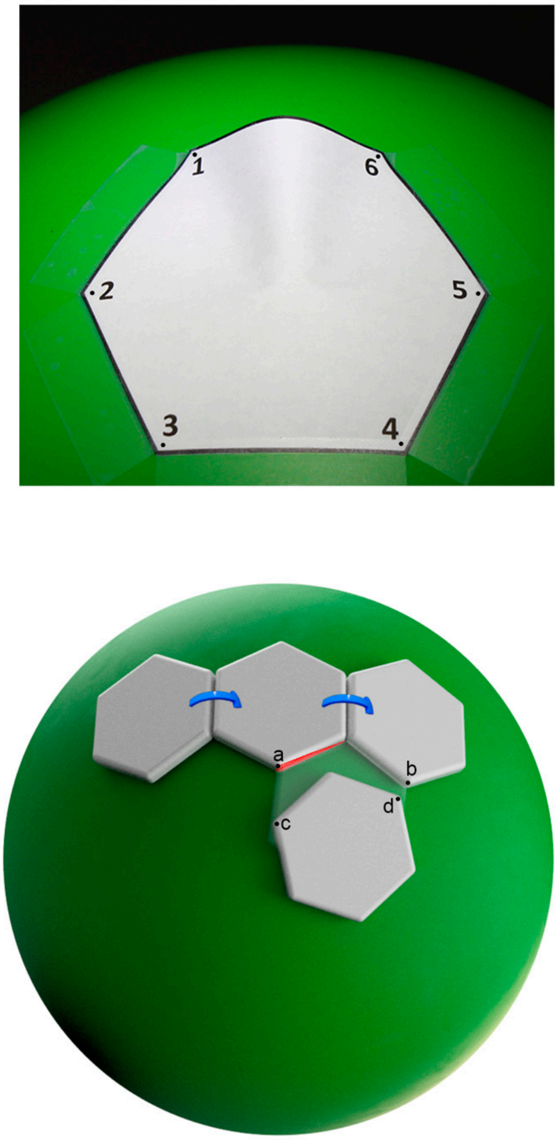

Supplemental Figure S2: Approaches tested to build a hexagon-based sphere. (A) A regular paper hexagon can be taped onto the surface of a balloon. If sides are attached to the surface in sequence, the last remaining side (1–6) is shorter than all other sides. An obvious paper bulge becomes visible. If a new hexagon is connected adjacent to side 1–6, the new hexagon must be smaller and will have an even smaller final side on its far side. This solution is not adequate to build a hexagonal lattice. (B) A second solution consists of adjacent hexagons that are allowed to rotate along connecting hinges (blue arrows). We refer to this method as the "cylinder" solution. The relative angular change of adjacent hexagon planes brings points (a) and (b) closer together. As a result, a new hexagon cannot fit into this space as the distance between points (c) and (d) is longer than the distance between (a) and (b). If we force the fit (overlap in red), immerging tile spaces are even smaller. This in turn generates increasingly more dramatic overlaps as more hexagons are added.
